# Future trends in synthetic biology in Asia

**DOI:** 10.1002/ggn2.10038

**Published:** 2021-03-05

**Authors:** Ning Mao, Nikhil Aggarwal, Chueh Loo Poh, Byung Kwan Cho, Akihiko Kondo, Chenli Liu, Wen Shan Yew, Matthew Wook Chang

**Affiliations:** ^1^ NUS Synthetic Biology for Clinical and Technological Innovation (SynCTI) National University of Singapore Singapore Singapore; ^2^ Synthetic Biology Translational Research Program and Department of Biochemistry, Yong Loo Ling School of Medicine National University of Singapore Singapore Singapore; ^3^ Department of Biomedical Engineering National University of Singapore Singapore Singapore; ^4^ Department of Biological Sciences, and KI for the BioCentury Korea Advanced Institute of Science and Technology Daejeon South Korea; ^5^ Graduate School of Science, Technology and Innovation, and Engineering Biology Research Center Kobe University Kobe Japan; ^6^ CAS Key Laboratory for Quantitative Engineering Biology, Shenzhen Institute of Synthetic Biology, Shenzhen Institutes of Advanced Technology Chinese Academy of Sciences Shenzhen China

**Keywords:** biotechnology, genomics, genetics or genomics, genetics or genomics, synthetic biology

## Abstract

Synthetic biology research and technology translation has garnered increasing interest from the governments and private investors in Asia, where the technology has great potential in driving a sustainable bio‐based economy. This Perspective reviews the latest developments in the key enabling technologies of synthetic biology and its application in bio‐manufacturing, medicine, food and agriculture in Asia. Asia‐centric strengths in synthetic biology to grow the bio‐based economy, such as advances in genome editing and the presence of biofoundries combined with the availability of natural resources and vast markets, are also highlighted. The potential barriers to the sustainable development of the field, including inadequate infrastructure and policies, with suggestions to overcome these by building public‐private partnerships, more effective multi‐lateral collaborations and well‐developed governance framework, are presented. Finally, the roles of technology, education and regulation in mitigating potential biosecurity risks are examined. Through these discussions, stakeholders from different groups, including academia, industry and government, are expectantly better positioned to contribute towards the establishment of innovation and bio‐economy hubs in Asia.

## Graphical abstract

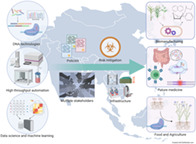

## 1 INTRODUCTION

With more than two decades of development in research and technology translation, synthetic biology is progressively realizing some of the promises in a variety of highly anticipated applications, such as healthcare, food and agriculture, and manufacturing.^1^, ^2^ The economic contribution from synthetic biology‐enabling or ‐enabled technologies is growing fast, at a projected compound annual growth rate (CAGR) of 28.8%, expected to reach a market size of US$18.9 billion by 2024.^3^ In order to further promote a strong growth of synthetic biology and the broader bio‐based economy, at least 50 governments have put forward long‐term strategies or groups of policies to invest resources and build talent pools.^4^ For instance, the research roadmap by the Engineering Biology Research Consortium (EBRC) delineates a comprehensive set of technical themes and application sectors as a guide for funding agencies and researchers.^5^ The UK has also established a bioeconomy strategy to build a collaborative research network, an expert workforce and a supportive business environment to catalyse biotechnology translation.^1^, ^6^, ^7^

Although significant research, entrepreneurship, and investment activities in synthetic biology have been highly concentrated in the US and the UK,^7^ there is also growing interest and determination in developing the bio‐based economy in Asia, as witnessed by the increasing investment in research and development^8^, ^9^, ^10^ and policy support at the national level.^11^, ^12^, ^13^ Workshops have been recently convened in Singapore by stakeholders from academia, industry, and government in Asia, and by the Asian Synthetic Biology Association (ASBA) to discuss the status of synthetic biology research ‐ vis‐à‐vis advancement and bottlenecks ‐ and potential risks and challenges to regulatory bodies. Through the workshops, representatives from different groups achieved better understanding of the technology and consensus regarding multilateral collaborations to effectively advance synthetic biology research and technology translation in Asia.

In this Perspective, we recount the main topics discussed during the workshops and give a review of the latest developments in synthetic biology research and applications, with highlights of noteworthy work from Asia (particularly East, South and Southeast Asia) and discussions on collaboration and regulation. First, the current state‐of‐the‐art in synthetic biology research and technology translation is reviewed from the perspectives of enabling technologies, and contextualized within three broad application sectors, respectively. Following that, a summary of the discussions on effective collaborations and regulations to mitigate biosecurity risks is presented. Some of the quotes and recommendations from the workshops are cited in Box 1. Collectively, the insights and recommendations from the workshop participants may facilitate a sustained development of synthetic biology research and applications in Asia.

Box 1 **Quotes from the workshops that underscore the main themes of the Perspective. The quotes are not attributed as the workshops were held under Chatham House rules to promote free and open discussions**

1. On the design‐build‐test‐learn (DBTL) cycle of synthetic biology:

“*In the near future, workflow for a biological engineer will no longer be limited by the pace of fabrication but instead by their ability to analyze circuit behavior and incorporate the data into the next design cycle*.”

2. On next‐generation biomanufacturing driven by synthetic biology:

“*Leverage synthetic biology not to replace existing systems, but to integrate, augment, advance and integrate into existing systems … and to make it better*.”

3. On future medicine driven by synthetic biology:

“*Therapeutic products used to be focused on individual molecules – now we are looking at whole organisms for clinical use*.”

4. On the importance of multi‐stakeholder engagement in driving innovation in synthetic biology:

*“Decision makers need to be informed of the technologies to make the right policies*.”

5. On the importance of dialogue on biosecurity at the global level:

*“Global collaboration requires global harmonization of understanding… and regulation*.”

## 2 SYNTHETIC BIOLOGY‐DEFINITION AND STAKEHOLDERS

Synthetic biology is the application of engineering principles, such as standardization, decoupling, and abstraction, to biology in order to develop biological systems with novel functionalities.^14^ It is a hybrid of both engineering and biological disciplines wherein “rewiring” of the naturally occurring biological circuitry, be it a gene or protein, is performed to achieve the logical form of cellular control for desired applications. The constituents of synthetic biology are widespread (some of them are summarized in Table 1) and there is considerable overlap with other fields, such as molecular biology and genetic engineering. The scope of synthetic biology is ever‐broadening due to the rapid progress in its enabling technologies, including DNA reading and writing, high‐throughput automation and data science. As such, the products of the field have evolved from simple genetic logic circuits to miniaturized genomes, expanded genetic code, reprogrammed metabolic pathways and autonomous multi‐layer circuitry across multiple species of microbes.

**TABLE 1:** Key constituents of synthetic biology. Grey shading denotes the constituent in use in the indicated sector

Constituents	In use in academia	In use in industry
Genetic parts, for example, promoters, terminators, RBS		
Broad‐host range plasmids		
Genome miniaturization		
Metabolic engineering		
DNA sequencing		
Codon optimization		
DNA data storage		
Self‐organizing multicellular structures		
Protein engineering		
CRISPR‐Cas		
Multiplexed genome editing		
High‐throughput screening and automation		
Synthetic chromosomes		
Microbiome engineering		
DNA assembly		
Gene drives		
Bioprospecting		
Cell‐free systems		
Xenobiology		
Genetic logic circuits		
Modelling and machine learning		
DNA synthesis		
Directed evolution		
Synthetic organelles		

Due to the inherent multidisciplinary and application‐oriented nature of synthetic biology, cooperation and mutual understanding of the various aspects of the field between various stakeholders is required to further progress and realize the full potential of the field. These stakeholders include research scientists and engineers who conceptualize, construct, and evaluate engineered biological systems. Among the stakeholders, biotechnology entrepreneurs and industry leaders can enable the translation of these engineered systems, resulting in the emergence of disruptive new businesses. Finally, policymakers can develop a governance framework for responsible research and risk mitigation to steer the development of synthetic biology to meet societal goals.

### 2.1 Honing the tools and mining for more resources in Asia

Enabling technologies are instrumental in making synthetic biology research possible. These broadly include DNA reading, writing and editing technologies, biomolecular and host engineering technologies, and data science. The continued development of these enabling technologies is imperative for the advancement of synthetic biology. Consequently, a number of Asian countries have recently established state‐sponsored research programs, national institutes and academia‐industry collaborations to drive technological innovations for the advancement of synthetic biology.^15^ Here we review some of the most important developments in enabling technologies of synthetic biology in Asia.

#### 2.1.1 DNA reading, writing and editing

A significant reduction in the cost of DNA reading and writing in the last few decades has been the most powerful driving force of synthetic biology development. Since the completion of the Human Genome Project, DNA sequencing and synthesis technologies have advanced rapidly with the per‐base cost of DNA sequencing reducing by four orders of magnitude,^16^ while the cost of sequencing and synthesizing a human genome has come down to US$1000 and US$6000, respectively.^17^

In Asia, there has been an increase in the penetration of markets by leading DNA synthesis and sequencing businesses, underscoring the growing demand for synthetic biology‐enabling tools in the region. Integrated DNA Technologies (IDT), a major player in custom DNA synthesis, opened a manufacturing facility in Singapore in 2013 to provide expedited delivery of its synthetic biology products to the Asian‐Pacific markets.^18^ It is one of the only two manufacturing facilities of IDT outside North America. In September 2020, IDT announced the establishment of a business entity in China to better support the region.^19^ Twist Biosciences, a US‐based rapidly growing provider of synthetic DNA and next‐generation sequencing (NGS) services, announced strategic partnerships with local distributors in Japan, South Korea, Hong Kong and India in 2018 to better serve the synthetic biology communities in these geographies.^20^ Thermo Fisher expanded its range of genome editing and DNA synthesis products available in China in 2018 resulting in the country becoming the biggest non‐US market for Thermo Fisher.^21^ Among the regionally grown companies, perhaps one of the largest in DNA sequencing is Beijing Genomics Institute (BGI) based in China. The company, funded by the Chinese Development Bank and powered by its proprietary NGS service, sequenced 1% of the human genome for the International Human Genome Project, co‐sponsored the 1000 genomes project and developed a catalogue of more than 1000 genomes of the human gut bacteria,^22^, ^23^ findings from which will empower the synthetic biology community to embark on ambitious projects. In 2017, BGI announced the creation of an institute of synthetic biology which will focus on DNA storage, bio‐manufacturing of natural materials, and medical genome editing.^24^

The best manifestation of the advances in DNA technologies and the growth of industries in this sector is the synthetic yeast genome (Sc2.0) project,^25^, ^26^ and by further extension, the Human Genome Project ‐ Write.^27^ In Sc2.0, among the 16 yeast chromosomes currently being synthetically reconstructed, 7 of them involved Asian universities and companies. Three of the chromosomes have been successfully reconstructed in China, and one of the chromosomes nearing completion in Singapore. BGI is involved in the construction of three of these chromosomes, either alone or in partnership with other universities.^25^

To achieve de novo genome assembly of higher complexities, further advancement in DNA reading, writing, and editing is required.^17^, ^27^ To this end, multiple emerging technologies and techniques are being developed, such as single‐molecule sequencing platforms (eg, Pacific Biosciences and Oxford Nanopore)^16^, ^28^ and enzymatic DNA synthesis (eg, Molecular Assemblies, DNA Script and Illumina).^29^, ^30^, ^31^ Last but not least, as a quality control measure as well as functional knock‐in/out, precision genome editing tools, such as CRISPR‐Cas and base editors, are continuously being improved with the demonstration of precision base‐editing techniques that do not incur double‐strand breaks^32^, ^33^, ^34^, ^35^ and multiplexed base editing.^36^, ^37^ Although the novel DNA reading and writing technologies are being developed primarily in the US and UK, exemplified by the leading startups being situated in these regions, Asia has seen substantial improvement in the DNA editing technologies, particularly CRISPR‐Cas, as a result of a dramatic increase in the government investment.^38^ China is now close behind the US in the number of recent CRISPR‐Cas patents, followed by Japan and South Korea.^39^ These countries are actively exploring the use of CRISPR‐Cas for medical and agricultural applications, with China leading the pack in the latter.

With the continuous evolution of DNA technologies in reading, writing, and editing, we will be better equipped to design, modify and build genomes of any organisms of interest. In addition to genome editing and synthesis, the latest DNA technologies will also fuel the burgeoning field of DNA data storage, which has the advantage of superior data density and durability.^30^, ^40^, ^41^

#### 2.1.2 High‐throughput platforms for molecular and host engineering

The advanced DNA technologies have empowered the next layer of engineering, namely biomolecular and host engineering. Within the last two decades, there have been demonstrations of intricate genetic circuits^42^, ^43^, ^44^, ^45^, ^46^ and significant effort has been put in, which has improved the predictability of the engineering process. For instance, genetic elements and system insulators were designed to reduce the context dependency of gene expression.^47^, ^48^, ^49^, ^50^ There are also data and model‐driven design tools^51^ to predict design outcomes from individual elements^52^, ^53^ to whole systems.^54^, ^55^ However, despite progress in design tools, the current status of synthetic biology applications is still largely a trial‐and‐error process involving “brute force” screening with multiple iterations of the design‐build‐test‐learn (DBTL) cycle, a central concept in traditional engineering that is now applied to synthetic biology.

To accelerate the DBTL cycle, integrated infrastructure with strong automation and computer‐aided design capabilities ‐ biofoundries ‐ were established in multiple research centers around the globe. A global alliance was formed in 2019^56^ to promote open source development of both software and hardware, and sharing of protocols, best practices, and standards. The vision is to enable rapid prototyping of engineered biological systems in an agile and reliable manner. Among the 28 public foundries of the Alliance, 8 are in Asia, with 4 in China, 2 in South Korea and 1 each in Japan and Singapore. The biofoundries, such as the London and Singapore biofoundries, provide cost‐effective access to high‐cost equipment and small‐scale prototype evaluation to other academic laboratories and companies.^57^

Establishment of public biofoundries requires substantial public investment, time, and trained personnel and thus, they are typically found in nations, particularly those in Asia, with a national synthetic biology program and a well‐defined bioeconomy roadmap. Biofoundries can significantly accelerate the engineering of biological systems by providing higher reproducibility and throughput and ease of sharing of standardized protocols. As other nations, such as India, formulate their bioeconomy strategies,^58^ biofoundries have the potential to be at the core of a nation's synthetic biology capabilities. The vital role that biofoundries play in synthetic biology is evident from some of the success stories from the existing biofoundries. For instance, in the eminent “10 molecules in 90 days” pressure test taken on by the Foundry at the Broad Institute,^59^ six of the challenged molecules or their close relatives were successfully produced within the given time constraint. In another study, the biofoundry at the University of Manchester produced 17 potential material monomers in 85 days.^60^ The London Biofoundry contributed to the rapid development and validation of automated SARS‐CoV‐2 clinical diagnostics^61^. Apart from a demonstration of capabilities, such drills also identified key bottlenecks in further accelerating the engineering process. These include gaps in computer‐aided design tools, time needed for DNA synthesis, and complicated analytical methods for product characterization and measurement.^59^

While academic research continues to improve the efficiency and reliability of the engineering methods and platforms, there are already a few commercial enterprises with business models centered upon developing customized enzymes or microbial hosts, such as the US‐based Ginkgo Bioworks and Zymergen. Such platform companies not only pioneered the technology translation, but also contributed towards advancing process automation, data curation, and production scale‐up.^62^ With strong in‐house capabilities of discovery, engineering and production, they will be the powerhouses in driving synthetic biology applications in a variety of sectors (to be discussed in later sections).

#### 2.1.3 Data science and machine learning

While the “build” step is empowered by advances in DNA technologies and automated liquid handling, the potential in speeding up the remaining three steps ‐ design, test, and learn ‐ lies in data science and machine learning algorithms. Driven by increasingly efficient and accessible sequencing capabilities, we are generating an explosive amount of data, in the form of genomic/metagenomic and transcriptomic information. When such genetic information is coupled with additional layers of profiling, such as metabolomics and proteomics, they offer powerful tools to understand the intricacy of biological systems, to discover novel enzymes, pathways, intercellular interactions, and ultimately aid in engineering design.^63^ For example, by mining the genome and profiling the transcriptome and metabolome of the UV‐resistant animal tardigrades, researchers in Japan were able to identify unique proteins that confer DNA protection against UV radiation.^64^ A Chinese group applied comparative genomic and transcriptomic analyses to the resurrection efforts of the plant *Selaginella tamariscina* and revealed genetic mechanisms of drought tolerance in plants.^65^ Similar multi‐omics approaches also led to the discovery of novel biosynthetic pathways from microbes isolated from Singapore's native environment,^66^, ^67^ as well as silent secondary metabolites in *Streptomyces* species.^68^ Such novel discoveries not only add to our understanding of the biological systems, but also enrich the available molecular tools for synthetic biology research and applications.

With more and more systemic biological data becoming available, the methods for extracting valuable information and enlightening biological designs from such datasets become the next technological challenge; at the same time, this has also created many opportunities for advances in machine learning.^69^, ^70^ For example, machine learning algorithms have been developed to predict promoter strength^71^ and natural product structures^72^, ^73^ from genome sequences, to predict function from molecular structure information,^74^ to aid the directed evolution of proteins,^75^, ^76^ to predict base editing outcomes,^77^ to accelerate metabolic pathway design and optimization,^78^, ^79^, ^80^ and to streamline analytical chemistry data processing during the test step of strain engineering.^81^, ^82^ These are just a few examples where machine learning has significantly improved the efficiency of otherwise tedious, labor‐intensive, or highly unpredictable procedures in engineering biology. Furthermore, combining machine learning with mechanistic systems modelling could enable us to realize the full potential of predictive biology.^83^ It is reasonable to expect increasingly valuable applications of machine learning algorithms in advancing the design, test and learning efficiencies.

In order to fully realize the value of the rich biological data source and unleash the power of machine learning, it is necessary to standardize the process and control the quality of data acquisition, database curation, as well as to establish protocols for proper sharing (eg, an extension of the Nagoya protocol to focus on data information sharing^84^). Essentially, new data infrastructure is required to gather biological data from disparate sources, and standardize, curate, and deploy the data for enhanced innovation. This objective has been incorporated into the synthetic biology strategies set out by the US (in EBRC 2019) and the European Union (in ERASynBio 2014). An excellent example of this is the Elixir project^85^ which coordinates European life science resources, including databases and software tools, among its 23 member countries to create a data infrastructure for the purpose of understanding the functioning of living organisms. Thailand also established the Thailand Bioresource Research Centre (TBRC) in 2015 to facilitate collection and sharing of biological materials and to serve as the foundation of their bioeconomy. Similar data infrastructure has been proposed by Japan in Bio‐strategy 2019^12^ to make use of the existing underutilized databases, and has been recognized as one of the key pillars for developing the bioeconomy. Other Asian nations also have a wealth of biological data scattered into various databases^86^, ^87^ but a dedicated facility to consolidate this is lacking. Thus, the need of the hour is to have a public‐funded initiative to generate a comprehensive and coherent data infrastructure with accessible data to promote the advances of data‐driven biological research and engineering. This initiative would be in line with the prevailing ethos of open innovation in synthetic biology.^88^

### 2.2 Unleashing the potential for applications in Asia

Among the broad application sectors, we centered our discussions around three major themes most relevant to Asia: (a) next‐generation biomanufacturing, (b) future medicine, and (c) food, agriculture and environmental applications. A summary of the advances made in these application sectors is provided in Box 2.

Box 2 **Recent advances in the application of synthetic biology in Asia**

1. Next‐generation biomanufacturing

- On‐demand production of chemicals using microbes
- Use of feedstock such as agricultural and energy crops, CO_2_, and agriculture and industrial wastes to produce bio‐based fuels and chemicals
- Production of novel functional materials
- Biofuel production by algae

2. Future medicine

- On‐demand production using living cells and cell‐free systems
- Precise genomic editing for inherited disorders using CRISPR‐Cas
- Engineered microbes and phages as therapeutics
- CRISPR‐Cas for sensitive detection of viruses

3. Food, agriculture and environment

- Genome‐edited crops for higher yield, resistance to disease and improved nutrition
- Microbial‐based fertilizers
- Biodegradable plastics produced by microbes
- Electronic waste recycling by engineered microbes

#### 2.2.1 Next‐generation biomanufacturing

Synthetic biology is driving a manufacturing revolution that explores alternative feedstocks and production processes, and further extends towards the development of products of better performance. In the 2018 BIO industry report, it was estimated that the global economic value of bio‐based production, including renewable chemicals and polymers, biofuels, enzymes and materials, reached US$355 billion.^89^ Among this immense volume of production activities, the next‐generation biomanufacturing driven by synthetic biology has brought in new advantages ‐ the improved efficiency and economic benefits, the potential to produce chemicals and materials of novel properties, and the sustainable “circular” model of production.

The preeminent driving force is the economic benefits of biomanufacturing of natural products and intermediates of high commercial value, such as fragrance, nutrients, and medicine.^90^ Conventionally, such molecules are commonly extracted from plants ‐ a costly and tedious procedure. Now with advanced DNA and biofoundry technologies described in the previous sections, we are able to “rewire” and “reassemble” identified natural biosynthesis pathways into microbial workhorses to produce (parts of) the desired chemicals in a reliable and “on‐demand” manner. Another motivation for the development of biomanufacturing is the use of renewable biomass feedstocks, which can be more eco‐friendly and significantly reduce the dependency on the fossil fuels.

Asian markets have seen a substantial increase in the production of bio‐based commodities, partially driven by the ready availability of biomass feedstock. Although China and India are among the biggest crude oil‐producing nations, Asia has a severe dearth of crude oil as it consumes 36% of the world's oil but produces only 10% with the production declining in recent years,^91^ thus relying on import from other regions. However, Asia is rich in biomass feedstock due to large‐scale agricultural activities in the region. Countries such as Malaysia, Indonesia, Thailand, China and India are among the biggest exporters of palm oil, rice, sugarcane, coconut oil and cassava.^92^ This has prompted numerous private entities to establish biorefineries in this region. For instance, Corbion, a global leader in the production of lactic acid and its derivatives from sugarcane, plans to open the world's largest lactic acid production site in Thailand by 2023.^93^ The Asia‐based Wilmar International and the US‐based Elevance established a joint venture biorefinery in Indonesia with a 180kMT capacity to utilize the local palm oil to produce novel specialty chemicals. A second biorefinery in Malaysia has also been proposed.^94^

Going beyond what is given by nature, synthetic biologists are also reinventing what can be produced by biological systems. Companies such as Spiber in Japan and Bolt Threads in the US have developed fermentation products derived from spider silk protein that are light‐weight and possess desirable mechanical properties that cater to different industries. Structural proteins are relatively under‐explored for synthetic biology applications. By tapping on the diversity and complexity of such proteins, functional materials that never existed before can be constructed.

Next‐generation biomanufacturing could also accelerate the transformation from the current “linear” economy into a more sustainable “circular” system. In pursuit of a paradigm shift from the petrochemical‐reliant industry, synthetic biology researchers and entrepreneurs are exploring alternative renewable feedstocks, as well as producing products that are more environmentally friendly. In Taiwan, researchers developed CO_2_‐based photosynthetic pathways to produce butyrate with the cyanobacteria *Synechococcus elongatus*.^95^ Similarly, other metabolic engineering demonstrations sought to improve the production efficiency of cyanobacteria for a myriad of high‐value chemicals.^96^ Advances in genetic engineering of microalgae also opened new avenues for photosynthetic production of biofuels and other valuable chemicals in eukaryotic algae.^97^, ^98^ In Thailand, part of the national policy on Bio‐, Circular and Green Economy encourages waste‐to‐energy projects, and microbial conversion of biomass waste to alkane and alkene‐based fuels is being explored as an exemplified opportunity.^99^

Although the manufacturing of bio‐based chemicals and fuels appears to be a promising alternative to conventional petrochemical‐based production, there are various challenges that need to be surmounted for the long‐term sustainability of this sector, especially in the Asian region. To support the growing bioeconomy, there will be a need to secure sustainable biomass supply by increasing the cultivation area for crops and lignocellulose biomass. However, increased use of habitable land for agriculture and food crops as feedstock for bio‐manufacturing is untenable in highly populous Asian nations. Use of lignocellulose biomass does not threaten food security but this feedstock requires pretreatment which is technologically demanding and costly and not yet commercially operative.^100^ The diversification of the feedstock to industrial or agricultural wastes and the enhanced utilization of photosynthesis could further drive cost reduction for the manufacturing of bio‐based chemicals and fuels.

Other challenges faced by researchers and businesses alike include synthesizing beyond nature. The well‐studied biosynthetic pathways can be exploited for commercial applications, but the real potential of “on‐demand” production of any molecules or designer materials will require the integrated advances in sequencing data generation and analytics to identify new biosynthetic pathways. These pathways can then be optimized by platform companies such as Ginkgo Bioworks, Genscript and Zymergen to improve the production efficacy of desired molecules. In parallel, it is also recognized that the combination of bio‐ and conventional chemical synthesis offers versatile solutions to some molecules that present challenging structures, unknown natural synthetic pathways, or are toxic to host cells.

Bottlenecks of technology translation often lie in production scale‐up. Projects originating from a research lab or an early‐stage startup often lack the resources to carry out pilot scale‐up studies. Moreover, the complexity of biological systems and the vast differences of industrial vs small‐scale bioreactor conditions often make the outcome of scaled production unpredictable.^101^ Further development of predictive models and better integration of modelling with experiments are important in making the scale up process more rational and predictable.^102^, ^103^ There are also companies such as Amyris that specialize in optimizing the scale‐up production of commodity molecules.^104^

Beyond feedstock availability and scale‐up, other considerations that influence bio‐manufacturing include industry infrastructure, including commercial‐scale biorefineries and supply‐chain for feedstocks, the location of biorefineries, and public policy. In contrast to the crop feedstock, infrastructure for agricultural and industrial waste and aquatic biomass is not well‐established, particularly in Asia. For instance, the development of new supply‐chains, construction time and deployment of large‐scale biorefineries have been identified as the primary barriers to India's biofuel target for 2030.^105^ The location of the biorefinery is also an important aspect that influences the economic viability of biomanufacturing as transportation of the feedstock to the biorefinery can have significant associated cost and carbon footprint. Tay et al showed that in a scenario where crops are transported to Singapore from neighboring Asian countries and converted into poly‐lactic acid in an existing refinery, the use of biomass feedstock was not economically competitive compared to the conventional petroleum‐based product, although it was more environmentally friendly.^106^ A pivot to the production of higher‐value chemicals may be a potential solution for nations such as Singapore which lack native biomass feedstocks. Lastly, a right mix of policies and incentives to support the biomanufacturing sector is needed to ensure sustainability and to close the capability gap and reduce the risks as further discussed below. However, care must be taken so that these policies and incentives ensure a manageable growth of the sector and do not create a substantial backlog of new projects.

#### 2.2.2 Future medicine

Applications in healthcare have contributed to the majority of synthetic biology translation and commercialization, and unsurprisingly, have attracted the lion's share of investments.^107^

Synthetic biology has propelled the advancement of the pharmaceuticals industry through the expansion of therapeutics manufacturing capabilities. Before the advent of recombinant DNA technologies, the majority of pharmaceutical products were limited to small molecules. In 1982, the first commercial synthetic human insulin was produced by engineered *E. coli*,^108^ replacing the historical standard of extracting it from animals. Ever since, recombinant DNA technologies have fueled the development and manufacturing of more biologics, such as protein and RNA‐based therapeutic products. In addition to macromolecules, synthetic biology also revolutionized the manufacturing of some small molecule drugs of high demand, such as the microbial production of artemisinin^109^ and cannabinoids^110^ to replace the conventional plant source. Going beyond the convention of cell‐based biologics manufacturing, pioneering studies using freeze‐dried cell‐free systems to produce therapeutic molecules in a portable, on‐demand manner ‐ from small molecules, short peptides, to antibody conjugates and vaccines^111^, ^112^ ‐ could potentially revolutionize the manufacturing and distribution model of pharmaceuticals.

In addition to the manufacturing of molecular therapeutics, synthetic biology also facilitates the development of a new class of cell‐based therapies and gene therapies. In 2017, the US Food and Drug Administration (FDA) approved the first chimeric antigen receptor (CAR)‐T cell therapy,^113^ and at the dawn of 2018, the first directly administered viral vector‐based gene therapy was also given the green light to market.^114^ Such examples of pioneering cell and gene therapies are the harbingers of more therapeutic innovations enabled by synthetic biology. Going beyond gene replacement therapies, which is the mainstream of the current gene therapy technology, the CRISPR‐Cas system has been developed into base‐editing tools that can bring us closer to precision gene editing for inherited diseases.^115^ With respect to cell therapies, CAR‐T cells are being enhanced to be safer,^116^ more versatile,^117^ and to be sourced and manufactured more robustly.^118^ The repertoire of engineered immune cells is also being expanded beyond T‐cells to include NK cells^119^ and macrophages.^120^ In addition to immune cells, engineered bacteria, single species or multi‐species consortia, are also being developed for skin, gastrointestinal, and other microbiome‐associated diseases,^121^, ^122^, ^123^, ^124^ and notably for systemic metabolic diseases, where the most advanced developments are already in human clinical trials for phenylketonuria.^125^, ^126^ Closely related are bacteriophages as highly potent and specific antimicrobials^127^; engineered phages were also developed as modulating agents to enhance the effect of chemotherapy in cancer treatment.^128^ It is envisioned that future smart medicine could come in the form of living cells that detect the diseased states and respond with therapeutic accuracy accordingly.^129^

In other medical applications apart from therapeutics, synthetic biology has also empowered new methodologies for diagnostics and prophylactics. For in vitro diagnostic applications, reaction mixes with nucleic acid sensors based on RNA toehold switch^130^, ^131^ or CRISPR‐Cas13/Cas12a^132^, ^133^, ^134^ were developed into rapid and sensitive diagnostic tools, with demonstrations in the detection of femto‐attomolar level viruses including dengue, zika, and most recently SARS‐CoV‐2 viruses.^135^, ^136^ There are already two companies established in the US to commercialize the technology, as these lyophilized reactions stored on paper‐like media have great potential in point‐of‐care diagnostics in the field or at low‐resource regions where manufacturing and cold‐chain logistics are hard to access. In addition to in vitro diagnostics, novel in vivo diagnostics have been developed using live engineered bacteria for the detection, reporting, and even recording of biomarkers associated with pathogen, inflammation and the use of antibiotics.^137^, ^138^, ^139^, ^140^ New methods for developing vaccines were also demonstrated in the examples of recoding the genome of influenza A virus with multiple premature termination codons,^141^ and genomic mining for interferon‐sensitive mutations in the design of influenza vaccines.^142^

Asia is set to play a vital role in harnessing synthetic biology for the development of novel therapeutics. With a rapid urbanization in the Asian countries leading to lifestyle changes and an ageing population, there has been an increase in the incidence of non‐communicable diseases such as diabetes, cancer and cardiovascular diseases.^143^ Combining this increase with the prevalence of numerous infectious diseases and a high population, Asia perhaps has the highest demand for therapeutics than any other geographical region. As such, numerous regional governments are promoting innovation in medicine to tackle the rising healthcare burden and fulfill the unmet local needs by providing capital, generous tax breaks, maturing infrastructure and streamlining regulations. For instance, due to its strong policy support, China is now ranked second in the world in cell and gene therapies with around 1000 clinical trials underway or conducted as of 2020,^144^ targeting cancers, HIV and hereditary diseases.^145^ India is also aiming to emerge as a hub for biopharmaceutical production with the government initiating an industry‐academic collaborative mission to enhance research and development, providing grants and exemption on service tax to support startups and introducing programs for personnel skill development.^146^

All the prior‐mentioned emerging medical applications still face different levels of technical challenges before they could be developed into mature products. Moreover, two general challenges facing the newer generation of cell and gene therapies are regulation and manufacturing. The therapeutics developed from biological systems are inherently more complex than molecular drugs, thus possessing higher uncertainty in safety profiles. The US FDA has drafted clinical trial guidelines for both cellular and gene therapies^147^ and live biotherapeutics,^148^ which facilitate regulatory reviews of such breakthrough new classes of therapeutics. Still, from the experiences of the regulatory bodies in the US, EU and Japan, the evaluation of cell and gene therapies inevitably undertook an adaptive approach, with higher tolerance to risk and uncertainties, but necessitating systemic post‐marketing surveillance measures.^149^ This requires highly skilled reviewers and well‐designed evaluation and surveillance framework, which the regulatory bodies in emerging markets of Asia will need to develop in‐house and learn from established systems. Even after the regulatory challenge, the commercial scale manufacturing of cell and gene therapies has proven to be a limiting factor. To circumvent the engineering challenge of a scalable, automated and robust good manufacturing practice (GMP) biotherapeutics processing system, strong private‐public partnerships will be essential and be most effective in advancing the entire field.^150^

#### 2.2.3 Food, agriculture and environmental applications

In addition to medical innovations and next‐generation biomanufacturing, synthetic biology is also driving technologies that attempt to solve the many challenges facing the environment and the growing population. The biofuel production from alternative sources discussed in previous sections is already one good example for resolving the mounting energy demand in a sustainable way. Here in this section, we collectively report on the discussions on food, agriculture and other environmental applications.

Significant investment has been put into food and agriculture technologies that could provide solutions to keep up with the pace of the growing global population and climate change. One perpetual theme is centered on boosting the yield of crops or improving the nutrient components in food products. Plant synthetic biology has witnessed progress in genome‐editing‐enabled precision breeding, which significantly reduces the time needed for selecting desirable traits.^151^ Among its wide applications in agriculture, there are three strategies in boosting crop yields, namely increasing carbon fixation efficiency, minimizing plant respiratory CO_2_ loss, and establishing nitrogen fixation mechanisms in non‐legume crops.^152^ On nitrogen fixation, there are genetic engineering methods for crop plants, cereal crops in most studies, to express heterologous nitrogenase genes or to form nodule‐like symbiosis similar to legume roots; others seek to engineer bacteria that are naturally associated with cereal crops to carry out nitrogen fixation.^153^, ^154^ In China, plant synthetic biologists are using genome editing to engineer aromatic rice and wheat resistant to powdery mildew.^155^ Rice, the staple food in Asia, has also been genetically modified to be resistant to bacterial blight.^156^ Currently, an array of genome editing technologies are being developed in 9 Asian countries to engineer food crops, primarily for disease resistance.^157^ These projects are still in the research and development phase and no product has reached the market yet.^157^ Notably, unlike the US and EU, Asian countries have not made their position clear on crops with edited genomes; therefore, it remains to be seen if these countries will follow in US's footsteps of approving engineered crops with no foreign DNA.^155^ It is likely that these countries might be in favor of cultivation of engineered crops once the indigenously developed products are ready.

The US‐based startup Pivot Bio has developed the first microbial‐based fertilizer for corn to replace the conventional chemical ones, and it is expected to simultaneously produce better crop yield and avoid chemical pollution to the environment. Additional examples in agri‐food applications include the “Golden Rice” project, where rice is engineered to contain provitamin A and is now being distributed in regions with high vitamin A deficiency burdens; yeast‐produced human milk oligosaccharides as supplement to formula milk, and various plant‐based or cell‐based alternative protein products to replace the energy and resource‐intensive animal meat.

Synthetic biology research also seeks to reduce the generation, and improve the recyclability, of waste. For example, as an alternative material to the fossil fuel‐based conventional plastics, biodegradable polymers can be produced through microbial fermentation,^158^, ^159^ such as polylactic acid (PLA), polybutylene succinate (PBS) and polyhydroxyalkanoates (PHA). Choi et al summarized key technology advancements in microbial production of a number of PHA monomers, including feedstock, host organisms, enzymes and metabolic pathways.^160^ The China‐based startup BluePHA, a team of iGEM alumni entrepreneurs, is among a few businesses in Asia venturing commercial production of PHA with microbial fermentation.^160^ On the other hand, more efficient breakdown of conventional plastics is another way to fight against the plastics pollution. Since the discovery of the poly(ethylene terephthalate) (PET)‐degrading bacterium *Ideonella sakaiensis* and the two key enzymes in 2016,^161^ there has been many efforts in evolving the enzymes for higher efficiency.^162^, ^163^ Apart from plastics pollution, synthetic biologists also seek to reduce and upcycle electronic wastes by exploring heavy metal and rare earth element reclamation and recovery through engineered microbes.^164^, ^165^, ^166^

Many of the research studies on environmental applications, such as alternative fuels, materials, or food production methods, face difficulty in translation into industrial processes or sustainable business models. To compete with the low cost of fossil‐fuel‐based products, significant technical advancement is necessary ‐ for example, sourcing for cheaper, non‐food feedstock that does not take up excessive land use, developing more cost‐effective pretreatment methods of lignocellulose and other biomass, as mentioned above, and developing consolidated downstream processing technologies that can also exploit high‐value byproducts.^167^, ^168^ In addition to technology breakthroughs, governments can put forward policy incentives to encourage the adoption of these new technologies, which will be discussed in the following section.

## 3 BUILDING A SUSTAINABLE GROWTH IN ASIA

Over the next few decades, against the backdrop of a booming economy, Asia will have to face a myriad of problems as it tries to tackle climate change along with a population that is rising rapidly in some countries while ageing in others. An increase in the incidence of diseases will require Asia to invest in innovation in medicine to provide affordable healthcare to its people. A rising population poses a challenge to Asia's agri‐food industry as it tries to cope with the high demand for food with limited availability of arable land. Depleting fossil fuels and rapid deterioration of the environment, exacerbated by climate change, will push Asia towards eco‐friendly alternatives. Although synthetic biology has the potential to provide solutions to these problems, the field has to be advanced further to mature these solutions and bring them into the society for the benefit of everyone.

Currently, there are multiple synthetic biology research centers established, including eight members of the Global Biofoundry Alliance, in the Asia region. A cross‐region organization called the Asian Synthetic Biology Association (ASBA) was also created to promote academic communications, collaborations and technology commercialization. According to BIO industry report, venture investment in therapeutic biotech companies in Asia topped US$2.6 billion in 2019, comparable to that in Europe and about 40% of the number in the US.^169^ A similar trend was observed in the agri‐food sector with five Asian countries in the top 10 investment markets for agri‐food receiving ~US$2.5 billion in the first half of 2020, compared to US$4.9 billion invested in the US out of the global US$10.5 billion.^170^ In recent years, global leading synthetic biology companies have started making inroads into the Asian markets. We do have the “seeds” for synthetic biology research and technology translation, but the next question would be how to cultivate a nurturing soil that supports the development of the “seeds into blossoms”.

In Asia, there are significant barriers to the application of synthetic biology that will have to be overcome before its full potential is realized. Some of these barriers have been alluded to in the previous sections such as the feedstock availability and infrastructure development for bio‐manufacturing and the lack of clear regulatory policies on crops with edited genomes. In the case of medicine, a major hurdle to the successful translation of novel therapeutics could be its cost, with gene therapies currently costing millions of dollars^171^ which is beyond the reach of common people. Poor intellectual property protection laws in some of the Asian countries is also a hindrance to technological innovation in synthetic biology with the Asian countries ranking in the bottom half of the 2020 International IP index, with the exception of Japan, Singapore and South Korea.^172^ The governments of Asian countries will have to upgrade their role from supporters to key enablers of innovation in synthetic biology in order to overcome these barriers.

Asian countries have a high disparity in levels of economic development, availability of natural resources, regulatory systems, and approach to investments. Yet, some of the challenges and solutions facing this region remain the same across the borders. Thus, it is imperative for these countries to build regional and cross‐border collaborations to leverage each country's strengths and work towards solving the challenges. Various inter‐governmental organizations, including the South Asian Association for Regional Cooperation (SAARC) and the Association of Southeast Asian Nations (ASEAN), already exist in Asia to enable active collaboration and promote economic and trade growth and may be well positioned to play an influential role in developing synthetic biology in the region.

Public‐private partnerships can also play an important role in spurring a sustained development of the field. Long‐term government funding is vital in supporting research work towards “grand challenges” at the early stage, for which the risk is too high for industry to accept.^173^ However, in addition to the long‐term investment in basic science, it is also important to have an effective mechanism to catalyze technology translation and commercialization, which is often best achieved through private‐public partnerships. Gauvreau et al discussed a “Key Innovation Technologies and Systems (KITS)” model^174^ as an ecosystem to propel research and technology deployment, where an integrated operation of research, industry, entrepreneurship and investment works to achieve sustainability of the ecosystem. In a close working relationship, the research projects in a public institute will be guided by key industry and societal needs, and an effective two‐way consultation ensures the products and processes being developed can be turned into viable business models. In addition, the ecosystem has capabilities to establish, and attract start‐ups, by having its own investment arms and necessary intellectual property support.

For technologies used for medical and high‐value chemical applications, the paths to commercialization are relatively clear; however, applications in the environmental and food sectors can be more challenging, constrained by the status of the market and economics. In such cases, government policies come in as an important lever to trigger the initial exploration of a valid commercialization model. For instance, in Su et al's review of biofuel policies in the US, the EU, and China, various policy supports were explored in the studied countries^175^: setting up clear objectives in targeted percentage usage of biofuel in mid‐to‐long terms; providing financial incentives (subsidies, cost‐sharing, tax incentives, public procurement, etc.) to reduce the barrier for the biofuel industry; and funding long‐term R&D projects to diversify biofuel feedstock, reduce production cost, and study overall emission and general environmental impacts. In parallel, carbon taxing and additional evaluation framework that incorporates negative impacts of fossil fuels in long term also help to “push” the demand for more sustainable alternatives. In another detailed study of the national biofuel policy in India,^176^ the authors emphasized many difficulties in achieving the renewable energy targets in the country; apart from the remaining technical challenges, it was also difficult to achieve a coordinated implementation of the biofuel policies at the federal and state levels, accountable long‐term stewardship by multiple ministries (eg, the Ministry of Energy, the Ministry of Agriculture, the Ministry of Finance, among others), and importantly, legal enforcement. Nevertheless, government support in the form of “technology push” and/or “market pull” policies^4^ is essential to de‐risk technologies for environmental applications, and make them more attractive to industry and investors who will subsequently explore potential sustainable business models. Equally important are frameworks and mechanisms for collaborative governance and performance evaluations.^4^ To come up with these policy frameworks, it is important to engage stakeholders from academia, multiple government functions and industry to achieve a common understanding of the objectives, the technology, as well as the market.

## 4 MITIGATING RISKS

It is a consensus that synthetic biology is a dual‐use technology, which not only has the potential to bring benefits to the society, but also has inherent risks of being misused. Entering 2020, the COVID‐19 pandemic again reminded us of the importance of biosecurity ‐ when a new pathogen emerges, it brings catastrophic damage to public health and the economy that has no regard for borders. In the future, as the technology to engineer biological systems becomes more accessible, the risk of any deliberate or accidental release of a pathogen or an engineered organism will also increase.^177^ Moreover, the growing reliance on biological data, especially sequence information, in medical and manufacturing applications makes future bio‐based industries prone to “bio‐hacking” through data breaches.^84^ While the synthetic biology community is making relentless efforts to continue to advance synthetic biology, the discussion on regulations also needs to be expanded and deepened to mitigate the risks that may arise as the field progresses.

Risk mitigation measures should adopt a multifaceted approach leveraging technology, regulation, and education. Various biocontainment methods are the first line of barrier preventing accidental release of pathogenic or engineered organisms. These can include physical biocontainment infrastructure design, as well as engineered biological systems (eg, auxotrophies, kill switches, xenobiological firewalls) that can be multilayered to limit the survival and spread of engineered organisms.^178^, ^179^ In addition to biocontainment measures, public policies and institutional oversight are important to prevent potential intentional misuses of synthetic biology. In this regard, a combination of both top‐down (from government and funding agencies) and bottom‐up (from research groups and research institutes) oversights should be established; at the same time, it is critical to strike a balance in mitigating risks without falling into over‐regulation, thus necessitating multi‐stakeholder conversations.^177^, ^178^, ^180^ Policy makers need to collaborate with synthetic biology practitioners such that they are informed about the technology's risk and benefits and can come up with the right policies to mitigate risks effectively. At the same time, synthetic biologists need to listen to public concerns and brainstorm collectively with experts in the fields of public health, cybersecurity, defense and bioethics, on strategies to minimize risk, and measures to identify and counter potential biosecurity events, should they happen. It is recommended to have such conversations early, so that risk assessment and mitigation measures do not fall behind technological advances.^178^ In Asia, a group of biotechnology experts carried out a biosecurity risk assessment in some of the Asian countries and found that although the countries possessed expertise in the area and well‐established capabilities to jointly investigate and respond to threats, there was inadequate legislation addressing biosecurity and no common understanding of biosecurity among the various stakeholders.^180^ This is likely to improve in the coming years as the countries become increasingly focused on deliberate biological threats, as observed in a recent multilateral biosecurity dialogue.^181^

In addition to biocontainment and regulations, education is an important way to mitigate risks by raising biosecurity awareness among researchers and the public. In the ethics modules of universities, and the code of conduct trainings for researchers, it is helpful to include the discussions of the dual‐use character of synthetic biology.^178^, ^179^ A targeted exercise established by the EBRC, the “Malice Analysis” workshops,^182^ gathers graduate students and researchers in the engineering biology community to practice their abilities to identify potential misuse of synthetic biology research, and to come up with mitigation plans accordingly. In the same vein, appropriately engaging the public on risk‐benefit discussions can have the benefits of conveying the message that the researchers and governments have carefully considered the risks of synthetic biology, and preventing potential future backlash in public opinions.^178^

Be it promoting technology advancement as a field, or setting up regulations to mitigate risks, regional and global collaborations are essential. Each country may have its specific problems and interests to invest in, such as the issue of tropical infectious diseases facing Southeast Asian countries. As a result, each country may prioritize development of technologies for solving its specific issues, and therefore will also have different levels of risk tolerance associated with applying new technologies.^177^ Currently, the standards for biosafety and biosecurity regulations are highly variable even within a single country.^183^ Experts are calling for more regional and even global harmonization and collaborations.^177^, ^178^, ^184^ For instance, in the face of a novel pathogen, coordinated real‐time communication and data sharing across borders will make biosecurity surveillance and response more effective.^184^ In order to achieve this, it is important to have researchers and regulators from different countries align their understanding of the benefits and risks of synthetic biology, and lay out common frameworks for synthetic biology regulations and biosecurity surveillance. One good example is the global harmonization effort by the International Gene Synthesis Consortium, which is establishing a standardized synthetic DNA screening mechanism and working with multiple stakeholders to implement this as a global norm to safeguard against misuses of synthetic DNA.^185^ Regional and global forums and working groups, such as SAARC and ASEAN, provide good opportunities for multilateral strategic planning, and facilitate the establishment of concerted biosecurity surveillance, preparedness and response mechanisms.

## 5 CONCLUSION AND RECOMMENDATIONS

A number of topics of synthetic biology were reviewed in this article, including research advances and technology translation, as well as mechanisms for future investment, growth, and regulations. We summarize some of the key consensuses and recommendations to the field, especially in Asia, through the lens of the workshops in Singapore: this is a collection of opinions from a diverse group of expert practitioners and thought leaders.

- Significant investment and technology advancement in DNA synthesis, computer aided design and process automation, biological data science and machine learning are critical to further enable synthetic biology research and accelerate the DBTL cycle.
- Biological sequence data are valuable information and central to synthetic biology applications, and their collection, curation and sharing processes should be streamlined and harmonized globally, with robust data security surveillance.
- Applications in various sectors have achieved different levels of commercialization; past experiences encourage early‐stage R&D project researchers to have the “end product” in mind and work closely with industry partners to develop the associated downstream processing, production scale‐up, and economic or business models to effectively fulfil unmet needs.
- To facilitate rapid technology translation and deployment, integrated research‐development‐investment (R&D&I) ecosystem models are worth exploring, where effective private‐public partnerships can be established.
- In addition to long‐term investment in the development of science and technology, governments should also utilize “push‐and‐pull” policy instruments to facilitate the adoption of bio‐based production, especially when such businesses face daunting competition from fossil fuel‐powered industries.
- Synthetic biologists and policy makers should engage multiple stakeholders (public health, data security, defense, economy, and the public) to come up with strategies and regulations for biosecurity surveillance, risk mitigation and effective response mechanisms.
- Regional and global collaboration and standard harmonization are essential in advancing synthetic biology as a field and bolstering biosecurity defense.

With decades of research and key technology advancements, we are witnessing some initial success in technology translation and commercialization of synthetic biology, particularly in next‐generation biomanufacturing and medical applications. Additional developments are necessary to realize the full potential of synthetic biology in not only industrial production, but also future smart medicine and environmental applications. We are optimistic that with the prior recommended actions and initiatives, strong collaborative synthetic biology R&D&I clusters can emerge in Asia and contribute to the sustainable growth of the global bioeconomy.

## AUTHOR CONTRIBUTIONS

**Ning Mao:** Conceptualization; investigation; writing‐original draft; writing‐review and editing. **Nikhil Aggarwal:** Conceptualization; investigation; writing‐original draft; writing‐review and editing. **Chueh Loo Poh:** Investigation; writing‐review and editing. **Byung‐Kwan Cho:** Writing‐review and editing. **Akihiko Kondo:** Writing‐review and editing. **Chenli Liu:** Writing‐review and editing. **Wen Shan Yew:** Investigation; writing‐review and editing. **Matthew Chang:** Conceptualization; funding acquisition; investigation; supervision; writing‐review and editing.

## CONFLICTS OF INTEREST

The authors declare no conflicts of interest.
